# Profound Autonomic Instability Complicated by Multiple Episodes of Cardiac Asystole and Refractory Bradycardia in a Patient with Anti-NMDA Encephalitis

**DOI:** 10.1155/2016/7967526

**Published:** 2016-04-17

**Authors:** Stephanie R. Mehr, Roy C. Neeley, Melissa Wiley, Avinash B. Kumar

**Affiliations:** ^1^Department of Anesthesiology, Vanderbilt University, Nashville, TN 37212, USA; ^2^Department of Anesthesiology, Division of Critical Care, Vanderbilt University, Nashville, TN 37212, USA

## Abstract

Anti-*N*-methyl-d-aspartate receptor encephalitis (anti-NMDARE) is autoimmune encephalitis primarily affecting young adults and children. First described about a decade ago, it frequently manifests as a syndrome that includes progressive behavioral changes, psychosis, central hypoventilation, seizures, and autonomic instability. Although cardiac arrhythmias often accompany anti-NMDARE, the need for long-term electrophysiological support is rare. We describe the case of NMDARE whose ICU course was complicated by progressively worsening episodes of tachyarrhythmia-bradyarrhythmia and episodes of asystole from which she was successfully resuscitated. Her life-threatening episodes of autonomic instability were successfully controlled only after the placement of a permanent pacemaker during her ICU stay. She made a clinical recovery and was discharged to a skilled nursing facility after a protracted hospital course.

## 1. Introduction

Anti-*N*-methyl-d-aspartate receptor encephalitis (anti-NMDARE) is a severe form of autoimmune encephalitis resulting from autoantibodies directed against GluN1 subunits of the NMDA receptor in the central nervous system. Dalmau and colleagues described this condition, which primarily affects young adults and children, for the first time as recently as 2007 [1, 2]. Patients frequently present with variable neuropsychiatric symptoms ranging from psychosis to seizures to catatonia, adding to the complexity of an early diagnosis. We obtained written consent from the patient's family to present a complex case of anti-NMDARE with catatonia, seizures, acute respiratory failure, and profound autonomic instability requiring aggressive interventions including multiple rounds of CPR and cardiac pacing in the ICU. We seek to focus on the ICU course of illness in this complex patient with a protracted hospital course.

## 2. Case Report

Our patient was a 31-year-old African American female with a 2-3-week history of acute behavioral changes, personality breakdown, sexual inappropriateness, and religious grandiosity. Her past medical history was significant for asthma, genital HSV, and polycystic ovarian syndrome. She was admitted to the psychiatry service for evaluation of her acute behavioral changes and cognitive decline. During her admission, she developed new onset grand-mal seizures and was transferred to the neurologic intensive care unit. She continued to have frequent seizures and began to develop worsening catatonia. The neurological workup included multimodal imaging and CSF studies that were positive for GluN1 antibodies, supporting a diagnosis of anti-NMDARE. Subsequent workup including CT scans of the chest, abdomen, and pelvis, ultrasound of the pelvis, and a PET scan was negative for a tumor etiology. Our patient did not have a tumor etiology.

As the frequency of seizures increased, her mental status deteriorated and she was intubated for airway protection on hospital day 18. She had escalating episodes of autonomic instability, manifested by episodes ranging from narrow complex tachycardia with heart rates in the 140–160 bpm to severe bradycardia induced by vasovagal maneuvers such as coughing, suctioning of the endotracheal tube, and defecation. These episodes were initially self-limited but over subsequent days necessitated active pharmacologic intervention including combination of multiple rounds of glycopyrrolate and/or atropine and low doses of epinephrine during the episodes.

The initial treatment of the bradycardic episodes was targeted at reduction of vagal stimuli and triggers for bradycardia. This included suppression of coughing episodes with intravenous fentanyl and premedication with inhaled lidocaine before endotracheal suctioning. Intermittent propofol and ketamine sedation were also attempted to decrease vasovagal triggers but no clinical efficacy was appreciated. An early tracheostomy (ICU day 5) was done to help alleviate the airway irritation, decrease IV sedation, and facilitate mobilization.

**Figure 1:**
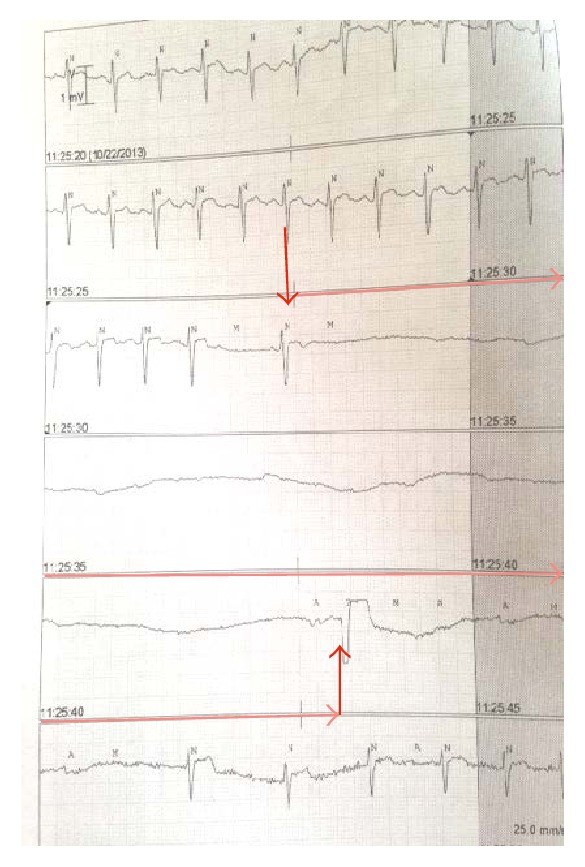
EKG strips with arrows outlining sudden progression of sinus tachycardia to asystole for more than 15 seconds before an escape beat and return of sinus rhythm (spontaneously).

**Figure 2:**
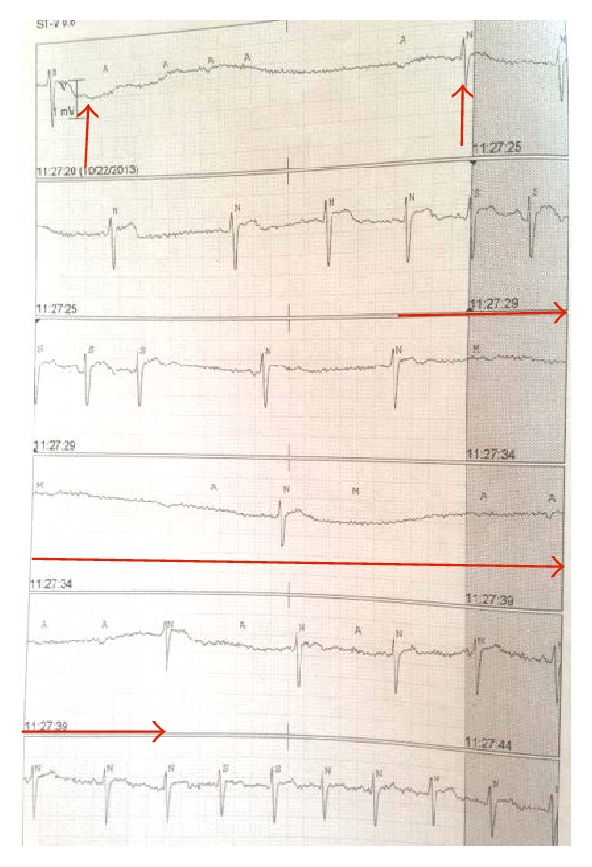
EKG strips with arrows outlining progression of severe bradycardia to transient asystole with return of sinus rhythm (spontaneously).

**Figure 3:**
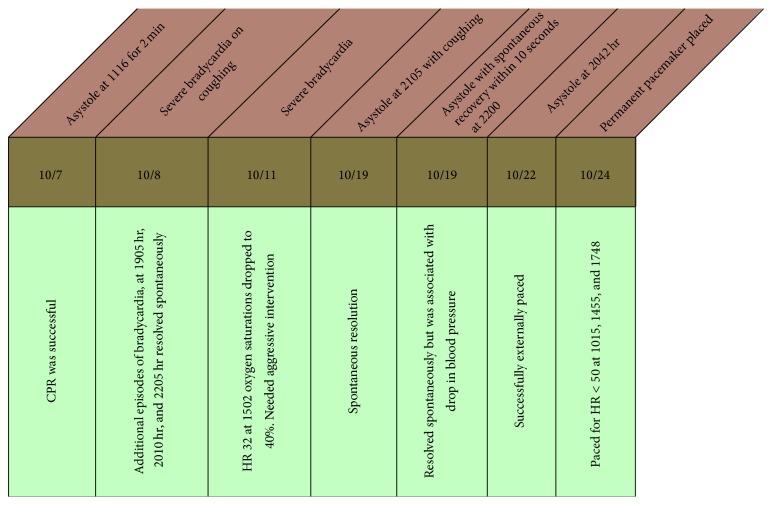
Timeline of autonomic instability and interventions in the ICU.

The patient continued to have two further episodes of severe bradycardia that progressed to cardiac asystole necessitating cardiopulmonary resuscitation. The period of asystole was recognized early and immediate initiation of CPR and chest compressions were carried out for two minutes with return of circulation. The EKG recordings showing progression to severe bradycardia are shown in Figures 1 and 2. She was transcutaneously paced during one episode to manage severe bradyarrhythmias as doses of glycopyrrolate and atropine (aliquots of 0.2 mg IV doses) were ineffective. Cardiac workup, including electrocardiogram, cardiac enzymes, and echocardiogram, was all within normal limits. Episodes of bradycardia and SVT occurred daily, until twenty days after the first episode of bradycardia, when a permanent pacemaker was placed. A snapshot of the episodes of autonomic instability is outlined in Figure 3.

**Figure 4:**
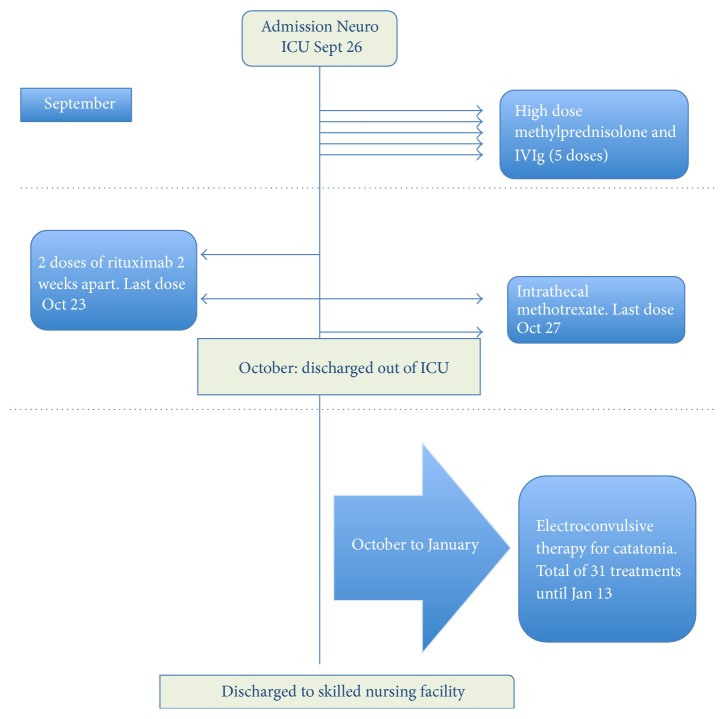
A brief snapshot of therapies and management strategies during hospitalization.

The patient received multiple therapies including high dose methylprednisolone and IVIg, immunotherapy with rituximab, intrathecal methotrexate, and an extended course of electroconvulsive therapy (ECT) for her profound catatonia. A visual snapshot of treatments offered is outlined in Figure 4.

The patient was discharged initially to a skilled nursing facility and then home. She made a good clinical recovery with the ability to take care of herself and family in due time.

Posthospital discharge interrogation of the pacemaker revealed 32 episodes of SVT (the longest lasting six and a half minutes) but no further severe bradycardia episodes beyond four days after pacemaker insertion.

## 3. Discussion

Our case illustrates the variable presentation and multisystem complexities that are involved in the care of this relatively new entity: anti-NMDA receptor encephalitis. From an epidemiological perspective, anti-NMDARE may be one of the more common causes of autoimmune encephalitis. The reported frequency of tumor or paraneoplastic association is also evolving as the literature accumulates about anti-NMDARE [3]. A recent large multicenter observational study of 577 patients found the association with a tumor etiology (teratomas) in less than 40% of cases [3]. Tumor etiology was ruled out in our patient.

We focus on the cardiovascular and autonomic instability in our case presentation. Bradycardia related to seizure activity and autonomic instability in anti-NMDARE has been previously reported [3, 4]. Central autonomic control of heart rate is surprisingly complex. Simplistically the resting heart rate is a function of the cardiogenic pacemaker and a balance between the bradycardiogenic parasympathetic system and the positive chronotropic sympathetic system. Mapping studies in animals have shown that several regions of the telencephalon, including the insula, anterior cingulate cortices, and amygdala, are involved in modulation of cardiac sympathetic and parasympathetic outflow. These connections eventually influence the preganglionic sympathetic and parasympathetic neurons of the heart [1]. Seizure induced activation can result in a synchronization of cardiac autonomic discharges with epileptogenic activity called the “lock-step” phenomenon and induces a lethal bradyarrhythmia or even asystole [4]. However in our case, not all bradycardia episodes were precipitated by vagal or epileptogenic stimuli. As the frequency of episodes escalated, several episodes of bradycardia did not have a clear precipitating factor and moreover these episodes were progressively more resistant to pharmacological interventions.

Class I indications for temporary pacemakers in the ICU include asystole, symptomatic bradycardia with hypotension and/or not responsive to atropine, and bifascicular blocks. Other common indications for temporary pacing include drug toxicities (digoxin toxicity, beta-blocker) and hyperkalemic bradycardia [5]. Frequently cardiologists will refrain from placing a pacemaker if the underlying mechanism of tachyarrhythmia-bradyarrhythmia is deemed episodic and/or related to the hyperacute phase of an illness. There is variability in the practice of implanting permanent pacemakers in noncardiac diseases especially if the indication is perceived to be reversible or time limited [6]. In case of anti-NMDARE, the autonomic instability can last for several weeks to months after the disease onset [1].

Anti-NMDARE patients frequently present to psychiatry services as the first point of contact. The psychiatric symptoms range from psychosis, auditory and visual hallucinations, aggression, anxiety, obsessive-compulsive behaviors, and depression to frank catatonia [7, 8]. The diagnosis is established by a positive serum or CSF sample screening for antibodies to the NMDA receptor subunit. Admission brain MRI scans have been reported as normal in as high as 70% of cases and EEG may show nonspecific slowing or slow continuous rhythmic activity during the later catatonic phase of illness [9]. There are small case series that describe a pattern of rhythmic delta activity (1–3 Hz) with superimposed bursts of rhythmic (20–30 Hz) beta activity that has been labeled “extreme delta brush” [10]. Multiple EEGs in our patient at the time of ICU admission were reviewed as delta waves with intermixed beta activity suggestive of an extreme delta brush pattern. This pattern was noticeably absent prior to the patient's transfer out of the ICU.

The key to an early diagnosis is often having a high index of suspicion in this younger patient cohort with new onset of neuropsychiatric symptoms combined with autonomic instability with or without seizures [3]. Prognosis of patients depends on early diagnosis, appropriate immunomodulatory therapy, and, in paraneoplastic cases, complete tumor removal. First-line immunotherapy is typically corticosteroids, intravenous immunoglobulins, or plasma exchange. Second-line therapy in resistant cases may necessitate using rituximab or cyclophosphamide and supportive care. Our patient underwent two courses of rituximab and intrathecal methotrexate as well.

The debilitating outcomes of anti-NMDARE are primarily from the persistent long-term cognitive deficits. It is worth noting that impairments in executive functions and memory can persist for an extended period of time even after clinical recovery and hospital discharge. Role of the NMDA receptors in the hippocampus is central to the postulated mechanism of cognitive and memory deficits. Connectivity to other areas such as the frontal cortices may play a role in the cognitive deficits as well [11].

Electroconvulsive therapy has been successfully tried in patients with severe psychiatric symptoms such as catatonia. Our patient underwent immunotherapy, immunosuppression, and long course of ECT prior to discharge. A schematic of the time course and treatments is outlined in Figure 3. Our patient had a recalcitrant course complicated by cardiorespiratory, psychiatric, and neurocognitive issues in spite of multimodal medical therapy until discharge from the hospital to a skilled nursing facility.

## 4. Conclusion

Early recognition, aggressive cardiorespiratory support in the ICU, and multistep pharmacological and supportive care by our multidisciplinary teams lead to a good outcome in our specific patient. Guidelines for early electrophysiological support in the setting of hemodynamic instability in anti-NMDARE are needed in the future.
